# Neonatal Sepsis: Mortality and Morbidity in Neonatal Sepsis due to Multidrug-Resistant (MDR) Organisms: Part 1

**DOI:** 10.1007/s12098-019-03106-z

**Published:** 2019-12-11

**Authors:** Chand Wattal, Neelam Kler, J. K. Oberoi, Anurag Fursule, Anup Kumar, Anup Thakur

**Affiliations:** 1 Department of Clinical Microbiology & Immunology, Sir Ganga Ram Hospital, New Delhi, India; 2 Department of Neonatology, Sir Ganga Ram Hospital, New Delhi, India

**Keywords:** Neonatal sepsis, MDR, Mortality, Morbidity, Financial burden

## Abstract

The major causes of emergence of multidrug-resistant organisms (MDRO) in neonatal sepsis include empiric antibiotic prescriptions, unregulated use of over-the-counter drugs, high incidence of healthcare associated infections (HAI), lack of awareness about antibiotic stewardship program and under staffing of neonatal intensive care units. In general, mortality due to MDRO sepsis is significantly higher as compared to non MDRO sepsis. Reported morbidities include prolonged use of total parenteral nutrition, need for central venous catheter, invasive ventilation, increased duration of hospital stay and neurologic sequelae.

## Introduction

Neonatal sepsis is a clinical syndrome characterized by systemic signs and symptoms of infection and is accompanied by bacteremia in the first month of life [1]. Early-onset sepsis (EOS) is defined as sepsis occurring in the first 72 h of life and that occurring beyond 72 h is defined as late-onset sepsis (LOS) [2]. As per World Health Organization (WHO), neonatal sepsis is the third most frequent etiology of neonatal mortality [3]. In the year 2013, a systematic analysis of global, national and regional causes of child mortality found neonatal sepsis to be the leading cause of neonatal deaths in India [4, 5]. The National Neonatal Perinatal Database network (NNPD, 2002–03) comprising of 18 tertiary care neonatal units across India reported sepsis (septicemia/meningitis) as the commonest cause of neonatal mortality, causing 23.4% of all neonatal deaths [6].

The pattern of the bacterial pathogens responsible for neonatal sepsis has changed temporally and geographically. There is a difference in the causative organisms for neonatal sepsis between the developed and developing countries [2, 7]. As per NNPD, *Klebsiella pneumoniae* and *Staphylococcus aureus* are the commonest causative organisms for EOS and LOS in India [6]. On the contrary, data from developed countries shows that gram-positive organisms are the predominant causes of EOS as well as LOS [2, 8].

The ability of bacteria to resist or to become tolerant to several structurally and functionally distinct drugs simultaneously is known as multidrug resistance [9]. Simpler definitions quote “multidrug-resistant organisms (MDROs) are labelled as such because of their in-vitro resistance to more than one antimicrobial agent”. On the other hand, definitions vary as per specific organism [10]. It is estimated that in India, 56,524 neonatal deaths each year are attributed to isolates resistant to first-line antibiotics [11].

## Multidrug Resistance: Global Picture

A recent point prevalence study – Antibiotic Resistance and Prescribing in European Children (ARPEC) was conducted in 226 hospitals (41 countries) which also included NICU data from our institute (Sir Ganga Ram Hospital, New Delhi). This survey showed that most commonly used regimen for neonatal sepsis was combination of ampicillin/amoxicillin/benzyl penicillin and aminoglycoside. It further reported that 40% pathogens isolated were resistant to first-line antibiotics prescribed by WHO [12]. Though this survey had paucity of data from low- and middle-income countries (LMICs), it provided important insights on emergence of antibiotic resistance. The resistance to first-line antibiotics in different WHO regions and is given in Table 1 [13].

**Table 1:** Resistance to first-line antibiotics in different WHO regions

Region as defined by WHO [14]	Resistance to Ampicillin (%)	Resistance to Gentamicin (%)
SEARO	97	83
AFRO	93	43
EURO	64	13

*SEARO* WHO South East Asian region; *AFRO* WHO Africa region; *EURO* WHO Europe region

Estimates of MDRO burden have also been reported from other countries. In a systematic review from five countries of South Asia (India, Pakistan, Sri Lanka, Bangladesh and Nepal) comprising of 109 studies, a high proportion of MDRO was reported. The pooled estimated data from hospital and community showed that *Klebsiella pneumoniae*, *E. coli* and *Acinetobacter baumannii* were multidrug resistant in 70.7%, 54%, 78.7% of isolates respectively [15]. A retrospective single centre study from Jordan evaluated 4 y data of 68 episodes of culture positive neonatal sepsis. Gram negative organisms were the commonest and 69% of these were multidrug resistant [16]. In another cohort study from Taiwan, conducted over 8 y, 1106 episodes of culture positive sepsis were reported. Of these, one-third were caused by gram negative bacilli and 70 (18.6%) were multidrug resistant [17]. A meta-analysis of 71 studies reported from China showed that 50% of gram negative organisms were resistant to third-generation cephalosporins [18].

## Multidrug Resistance: Scenario in India

A study of microbiological profile of *E. coli* from three NICUs in India which included 67 neonates with *E. coli* sepsis reported that majority of the isolates were resistant to cefotaxime (87%), ciprofloxacin (70%) and trimethoprim/sulfamethoxazole (76%). Phenotypic tests demonstrated that 87% of isolates were extended β lactamases (ESBL) producers and 6% were metallo β lactamases (MBL) producers. Nine isolates which were meropenem resistant possessed New Delhi metallo β lactamases-1 (NDM-1) [19]. In another study from Kolkata, carbapenem resistance in neonatal sepsis was evaluated over 5 y. Fourteen percent of isolates were carbapenemese resistant and in all of them, NDM-1 was identified [20]. Similarly, Chatterjee et al. observed that 56% of *Acinetobacter baumannii* isolated in blood culture were carbapenem resistant and 22% had NDM-1 [21].

A recent study, Delhi Neonatal Infection Study (DeNIS) reported clinical and microbiological data from three large tertiary care NICUs on 1005 culture positive cases [22]. Two-third of isolates were gram-negative. Commonest organisms isolated were *Acinetobacter baumannii* (22%) followed by *Klebsiella pneumoniae* (17%) and *Escherichia coli* (14%). There were high rates of multidrug resistance (resistance to any three of five antibiotic classes) in *Acinetobacter baumannii* (82%), *Klebsiella pneumoniae* (54%), and *Escherichia coli* (38%) isolates. In another cohort study from Delhi, multi-drug resistance rates in *Klebsiella pneumoniae*, *A. baumannii*, *E. coli*, *E. cloacae* were reported to be 65.4%, 71.4%, 78.7% and 66.0 respectively [23].

In a recent randomized controlled trial conducted at Sir Ganga Ram Hospital, New Delhi, out of 50 infants with gram negative sepsis, 54% isolates were MDROs. Fifty eight percent of *Klebsiella pneumoniae*, 62.5% of *Burkholderia cepacia* and 66.6% of *Acinetobacter baumanii* were multidrug resistant.

## Risk Factors for Development of Antimicrobial Resistance

Global consumption of antibiotics has risen by 36% from 2000 to 2010. BRICS countries (Brazil, Russia, India, China, and South Africa) contributed to 75% of this increase inspite of overall representation of only 40% of the world’s population [24]. In India, poor enforcement of regulations of over-the-counter sales of antibiotics, its low cost and an increase in consumption due to economic growth and prosperity are important factors implicated in rise of antibiotic resistance [24].

The major factors responsible for emergence and propagation of antimicrobial resistance (AMR) are overuse and empiric use of antibiotics, poor infection control in clinics and hospitals [25], lack of knowledge regarding organisms and its inherent antibiogram and rampant use of unreasonable fixed drug combinations [25–28]. Understaffing of NICUs are a major concern in resource limited developing countries [29]. Recent extensive use of antibiotics in agriculture has compounded the already existing problem of AMR. Antimicrobial growth promotors play a crucial role in this industry since they improve feed conversion, animal growth and reduce morbidity and mortality due to clinical and subclinical diseases [30].

## Outcomes in MDRO Sepsis

### Mortality

Various studies conducted globally have reported higher case fatality rates (CFR) due to MDRO sepsis. A study from Jordan reported that sepsis due to MDROs was associated with significantly higher mortality rate as compared to non-MDRO sepsis (60% *vs*. 13%) [16]. Similarly, another study from Taiwan reported mortality rate of 26.3% due to MDR *Acinetobacter baumanni* [17].

From India, the DeNIS study reported that the population attributable risk of mortality was 15.7% in culture-positive sepsis by MDRO *vs*. 12.0% in culture-positive sepsis by non-MDRO [22]. Another study from Delhi reported CFR of culture-positive and culture negative sepsis to be 23.0% and 6.8% respectively. The CFR among MDRO was higher than sensitive isolates. Among the neonates with MDR sepsis, only half survived [23]. In a recent data (2018–2019) from Sir Ganga Ram Hospital, New Delhi 64 culture positive extramural neonates cases had high mortality in MDRO as compared with non-MDRO [34.4% *vs*. 8%, *p* = 0.028, OR = 6.02 (C.I; 1.1–30.3)].

### Morbidities

In a study done in Taiwan, 376 episodes of gram negative bacteremia (GNB) were analysed. Underlying neurologic sequelae (22.9% *vs*. 13.4%), renal disease (12.9% *vs*. 1.3%), previous episode of bacteremia (35.7% *vs*. 23.5%), use of total parenteral nutrition (80% *vs*. 67.6%), use of central venous catheter (87.1% *vs*. 73.2%) were significantly high in MDR GNB as compared to non-MDR GNB cohort [31].

### Financial Implications

Though limited data exists on cost analysis, however, data from our institute (2010) from 59 episodes of culture proven sepsis (20 multidrug resistant isolates) showed prolonged duration of hospital stay in MDRO sepsis (27.6 d) *vs*. non-MDRO sepsis (20.7 d). The mean cost of therapy for MDRO sepsis was INR 4,99,840 *vs*. INR 180,592 for non-MDRO sepsis. On subgroup analysis of infants <1000 g birth weight, the mean cost of therapy in MDRO sepsis *vs*. non-MDRO sepsis showed further difference (INR 7,34,798 *vs*. 2,50,558) [32].

A study from USA reported escalation of cost of therapy due to ESBL *Klebsiella* outbreak in NICU to be 3,41,751 dollars, with major fraction of increase in cost due to health care provider time engaged in patient care [33]. Gandra et al. estimated the economic burden due to antibiotic resistance. Despite methodological limitation and difference in ways of estimation of disease burden, excess cost attributable due to infection caused by resistant organism *vs*. sensitive organism was much higher (Table 2) [34].

**Table 2:** Surplus expenditure in MDRO

Resistant Organism	Control	Range of Excess Cost
MRSA	MSSA	$695–$29,030
Vancomycin resistant *Enterococcus*	Vancomycin susceptible *Enterococcus*	$16,711–$60,988
Resistant *Pseudomonas aeruginosa*	Susceptible *P. aeruginosa*	$627–$45,256
Resistant *Acinetobacter baumannii*	Susceptible *A.baumannii*	$5336–$126,856
Multiple organisms	Susceptible	$9372–$18,990
ESBL producing Enterobacteriaceae	Non-ESBL producing Enterobacteriaceae	$3658–$4892

*ESBL* Extended β lactamases; *MRSA* Methicillin resistant *Staphylococcus aureus*; *MSSA* Methicillin sensitive *Staphylococcus aureus*

## Tackling Antimicrobial Resistance

Adherence to optimum hand hygiene practice is a cardinal step to prevent infection with MDRO. Antimicrobial stewardship programs help to optimize clinical outcomes, reducing untoward consequences of antibiotic use like toxicity and the emergence of resistance [35]. In a recent study in a single NICU, Jinka et al. found that implementation of antimicrobial policy based on microbiology information for neonatal sepsis resulted in increased use of first-line agents and reduced use of third-generation cephalosporins without effecting patient outcomes [36].

Regulation for over-the-counter drugs sale laid down by Central Drugs Standard Control Organization (CDSCO) has been a step to tackle AMR in India [37]. The Indian National Action Plan on AMR (2017–21) aims to gradually eliminate animal use of critically important antibiotics used on humans [38]. Other measures include optimizing antenatal care, intrapartum care, postnatal care and reducing use in suspected viral infections [15].

## Conclusions

Resistance is surging rapidly in LMIC countries. Infection with MDRO leads to higher mortality and morbidity in neonates. The economic burden is exponentially increased in MDRO sepsis. Risk factors for spread of MDRO include indiscriminate use of antibiotics, lack of hand hygiene, poor antibiotic stewardship and other socioeconomic factors including poor sanitation, lack of nurse patient ratio and overcrowding. Tackling spread of MDRO is an emergency and recommendations of WHO/CDC should be implemented with utmost sincerity in units.
